# Supplementary material for: Geometry-complete diffusion for 3D molecule generation and optimization
Source: Commun Chem. 2024 Jul 3;7:150. doi: 10.1038/s42004-024-01233-z (PMC11222514; doi:10.1038/s42004-024-01233-z)
Supplement: Supplementary file 2 — Supplementary Information [file 42004_2024_1233_MOESM2_ESM.pdf]

Supplementary Information for: Geometry-Complete Diffusion for 3D Molecule Generation and Optimization

Alex Morehead[1*] and Jianlin Cheng[1]

[1]Electrical Engineering & Computer Science, NextGen Precision Health, University of Missouri, Columbia, 65211, Missouri, USA.

*Corresponding author(s). E-mail(s): acmwhb@missouri.edu; Contributing authors: chengji@missouri.edu;

Keywords: Geometric deep learning, Diffusion generative modeling, 3D molecules

Appendices

A Supplementary Methods **3**
A.1 Expanded Discussion of Denoising 3
A.1.1 Geometry-Complete Denoising 3
A.1.2 GCPNet++ 3
A.1.3 Properties of GCDM 5
A.2 Expanded Discussion of Diffusion 6
A.2.1 Diffusion Models 6
A.2.2 Zeroth Likelihood Terms for GCDM Optimization Objective . 7
A.2.3 Diffusion Models and Equivariant Distributions 8
A.2.4 Training and Sampling Procedures for GCDM 8

B Supplementary Notes **10**
B.1 Broader Impacts 10
B.2 Training Details 10
B.3 Compute Requirements 10
B.4 Reproducibility 11

C Supplementary Results **12**
C.1 Property-Guided 3D Molecule Optimization - QM9 12

Appendix A Supplementary Methods

A.1 Expanded Discussion of Denoising

A.1.1 Geometry-Complete Denoising

In this section, we postulate that certain types of geometric neural networks serve as more effective 3D graph denoising functions for molecular DDPMs. We describe this notion as follows.

Hypothesis A.1. (Geometry-Complete Denoising)**.**

Geometric neural networks that achieve geometry-completeness are more robust in denoising 3D molecular network inputs compared to models that are not geometry-complete, in that geometry-complete methods unambiguously define direction-robust local geometric reference frames.

This hypothesis comes as an extension of the definition of geometry-completeness from Du et al. [1] and Morehead and Cheng [2]:

Definition A.2. (Geometric Completeness)**.**

Given a pair of node positions (x_i^t, x_j^t) in a 3D graph $\mathcal{G}$, with vectors $a_{ij}^t \in \mathbb{R}^{1\times3}$, $b_{ij}^t \in \mathbb{R}^{1\times3}$, and $c_{ij}^t \in \mathbb{R}^{1\times3}$ derived from (x_i^t, x_j^t), a local geometric representation $\boldsymbol{\mathcal{F}}_{ij}^t = (a_{ij}^t, b_{ij}^t, c_{ij}^t) \in \mathbb{R}^{3\times3}$ is considered geometrically complete if $\boldsymbol{\mathcal{F}}_{ij}^t$ is non-degenerate, hence forming a local orthonormal basis located at the tangent space of x_i^t.

An intuition for the implications of Hypothesis A.1 and Definition A.2 on molecular diffusion models is that geometry-complete networks should be able to more effectively learn the gradients of data distributions [3] in which a global force field is present, as is typically the case with 3D molecules [1]. This is because, broadly speaking, geometry-complete methods encode local reference frames for each node (or edge) under which the directions of arbitrary global force vectors can be mapped. In addition to describing the theoretical benefits offered to geometry-complete denoising networks, we support this hypothesis through specific ablation studies in Sections 2.1 and 2.3 of the main text where we ablate the geometric frame encodings from GCDM and find that such frames are particularly useful in improving GCDM's ability to generate realistic 3D molecules.

A.1.2 GCPNet++

Inspired by its recent success in modeling 3D molecular structures with geometry-complete message-passing, we parametrize p_{Φ} using an enhanced version of Geometry-Complete Perceptron Networks (GCPNets) that were originally introduced by

Morehead and Cheng [2]. To summarize, GCPNET is a geometry-complete graph neural network that is equivariant to SE(3) transformations of its graph inputs and maps nicely to the context of Hypothesis A.1.

In this setting, with ($h_i \in \mathbf{H}$, $\chi_i \in \boldsymbol{\chi}$, $e_{ij} \in \mathbf{E}, \xi_{ij} \in \boldsymbol{\xi}$), GCPNET++, our enhanced version of GCPNET, consists of a composition of Geometry-Complete Graph Convolution (**GCPConv**) layers $(h_i^l, \chi_i^l), x_i^l = \mathbf{GCPConv}[(h_i^{l-1}, \chi_i^{l-1}), (e_{ij}^{l-1}, \xi_{ij}^{l-1}), x_i^{l-1}, \mathcal{F}_{ij}]$ which are defined as:

$$n_i^l = \boldsymbol{\phi}^l(n_i^{l-1}, \mathcal{A}_{\forall j \in \mathcal{N}(i)} \boldsymbol{\Omega}_\omega^l(n_i^{l-1}, n_j^{l-1}, e_{ij}^{l-1}, \xi_{ij}^{l-1}, \mathcal{F}_{ij})), \tag{A1}$$

where $n_i^l = (h_i^l, \chi_i^l)$; $\boldsymbol{\phi}^l$ is a trainable function; l signifies the representation depth of the network; $\mathcal{A}$ is a permutation-invariant aggregation function; $\boldsymbol{\Omega}_\omega$ represents a message-passing function corresponding to the ω-th **GCP** message-passing layer [2]; and node i's geometry-complete local frames are $\mathcal{F}_{ij}^t = (a_{ij}^t, b_{ij}^t, c_{ij}^t)$, with $a_{ij}^t = \frac{x_i^t - x_j^t}{\|x_i^t - x_j^t\|}, b_{ij}^t = \frac{x_i^t \times x_j^t}{\|x_i^t \times x_j^t\|}$, and $c_{ij}^t = a_{ij}^t \times b_{ij}^t$, respectively. Importantly, GCPNET++ restructures the network flow of **GCPConv** [2] for each iteration of node feature updates to simplify and enhance information flow, concretely from the form of

$$\hat{n}^l = n^{l-1} + f(\Omega_{\omega, v_i}^l | v_i \in \mathcal{V}) \tag{A2}$$

to

$$\hat{n}^l = n^{l-1} \cup f((g_{e^\omega, v_i}^l, \Omega_{e^\omega, v_i}^l, \Omega_{\xi^\omega, v_i}^l) | v_i \in \mathcal{V}) \tag{A3}$$

and from

$$n^l = \mathbf{ResGCP}_r^l(\tilde{n}_{r-1}^l) \tag{A4}$$

to

$$n^l = \mathbf{GCP}_r^l(\tilde{n}_{r-1}^l). \tag{A5}$$

Note that here f represents a summation or a mean function that is invariant to node order permutations; $\cup$ denotes the concatenation operation; g_{e^ω, v_i}^l represents the binary-valued (i.e., [0, 1]) output of a scalar message attention (gating) function, expressed as

$$g_{e^\omega}^l = \sigma_{inf}(\phi_{inf}^l(\Omega_{e^\omega}^l)) \tag{A6}$$

with $\phi_{inf} : \mathbb{R}^e \rightarrow [0, 1]^1$ mapping from high-dimensional scalar edge feature space to a single dimension and σ denoting a sigmoid activation function; r is the node feature update module index; **ResGCP** is a version of the **GCP** module with added residual connections; and $\Omega_{\omega, v_i}^l = (\Omega_{e^\omega, v_i}^l, \Omega_{\xi^\omega, v_i}^l)$ represents the scalar (e) and vector-valued (ξ) messages derived with respect to node v_i using up to ω message-passing iterations within each GCPNET++ layer.

We found these adaptations to provide state-of-the-art molecule generation results compared to the original node feature updating scheme, which we found yielded sub-optimal results in the context of generative modeling. This highlights the importance of customizing representation learning algorithms for the generative modeling task at hand, since reasonable performance may not always be achievable with them without careful adaptations. It is worth noting that, since GCPNET++ performs message-passing directly on 3D vector features, GCDM is thereby the first diffusion generative

model that is in principle capable of generating 3D molecules with specific vector-valued properties. We leave a full exploration of this idea for future work.

A.1.3 Properties of GCDM

If one desires to update the coordinate representations of each node in $\mathcal{G}$, as we do in the context of 3D molecule generation, the **GCPConv** module of GCPNet++ provides a simple, SE(3)-equivariant method to do so using a dedicated **GCP** module as follows:

$$(h^l_{p_i}, \chi^l_{p_i}) = \mathbf{GCP}^l_p(n^l_i, \mathcal{F}_{ij}) \tag{A7}$$

$$x^l_i = x^{l-1}_i + \chi^l_{p_i}, \text{where } \chi^l_{p_i} \in \mathbb{R}^{1\times 3}, \tag{A8}$$

where $\mathbf{GCP}^l_{\cdot}(\cdot, \mathcal{F}_{ij})$ is defined to provide chirality-aware rotation and translation-invariant updates to h_i and rotation-equivariant updates to χ_i following centralization of the input point cloud's coordinates $\mathbf{X}$ [1]. The effect of using positional feature updates χ_{p_i} to update x_i is, after decentralizing $\mathbf{X}$ following the final **GCPConv** layer, that updates to x_i then become SE(3)-equivariant. As such, all transformations described above satisfy the required equivariance constraints. Therefore, in integrating GCPNet++ as its 3D graph denoiser, GCDM achieves SE(3) equivariance, geometry-completeness, and likelihood invariance altogether. Important to note is that GCDM subsequently performs message-passing with vector features to denoise its geometric inputs, whereas previous methods denoise their inputs solely using geometrically-insufficient scalar message-passing [4] as we demonstrate through our experiments in Section 2 of the main text.

A.2 Expanded Discussion of Diffusion

A.2.1 Diffusion Models

Key to understanding the contributions in this work are denoising diffusion probabilistic models (DDPMs). As alluded to previously, once trained, DDPMs can generate new data of arbitrary shapes, sizes, formats, and geometries by learning to reverse a noising process acting on each model input. More precisely, for a given data point $\mathbf{x}$, a diffusion process adds noise to $\mathbf{x}$ for time step $t = 0, 1, ..., T$ to yield $\mathbf{z}_t$, a noisy representation of the input $\mathbf{x}$ at time step t. Such a process is defined by a multivariate Gaussian distribution:

$$q(\mathbf{z}_t|x) = \mathcal{N}(\mathbf{z}_t|\alpha_t \mathbf{x}_t, \sigma_t^2 \mathbf{I}), \tag{A9}$$

where $\alpha_t \in \mathbb{R}^+$ regulates how much feature signal is retained and σ_t^2 modulates how much feature noise is added to input $\mathbf{x}$. Note that we typically model α as a function defined with smooth transitions from $\alpha_0 = 1$ to $\alpha_T = 0$, where a special case of such a noising process, the variance preserving process [3, 5], is defined by $\alpha_t = \sqrt{1 - \sigma_t^2}$. To simplify notation, in this work, we define the feature signal-to-noise ratio as $\mathrm{SNR}(t) = \alpha_t^2/\sigma_t^2$. Also interesting to note is that this diffusion process is Markovian in nature, indicating that we have transition distributions as follows:

$$q(\mathbf{z}_t|\mathbf{z}_s) = \mathcal{N}(\mathbf{z}_t|\alpha_{t|s}\mathbf{z}_s, \sigma_{t|s}^2 \mathbf{I}), \tag{A10}$$

for all $t > s$ with $\alpha_{t|s} = \alpha_t/\alpha_s$ and $\sigma_{t|s}^2 = \sigma_t^2 - \alpha_{t|s}^2 \sigma_s^2$. In total, then, we can write the noising process as:

$$q(\mathbf{z}_0, \mathbf{z}_1, ..., \mathbf{z}_T|\mathbf{x}) = q(\mathbf{z}_0|x) \prod_{t=1}^{T} q(\mathbf{z}_t|\mathbf{z}_{t-1}). \tag{A11}$$

If we then define $\boldsymbol{\mu}_{t\to s}(\mathbf{x}, \mathbf{z}_t)$ and $\sigma_{t\to s}$ as

$$\boldsymbol{\mu}_{t\to s}(\mathbf{x}, \mathbf{z}_t) = \frac{\alpha_{t|s}\sigma_s^2}{\sigma_t^2}\mathbf{z}_t + \frac{\alpha_s \sigma_{t|s}^2}{\sigma_t^2}\mathbf{x} \quad \text{and} \quad \sigma_{t\to s} = \frac{\sigma_{t|s}\sigma_s}{\sigma_t},$$

we have that the inverse of the noising process, the true denoising process, is given by the posterior of the transitions conditioned on $\mathbf{x}$, a process that is also Gaussian:

$$q(\mathbf{z}_s|\mathbf{x}, \mathbf{z}_t) = \mathcal{N}(\mathbf{z}_s|\boldsymbol{\mu}_{t\to s}(\mathbf{x}, \mathbf{z}_t), \sigma_{t\to s}\mathbf{I}). \tag{A12}$$

The Generative Denoising Process. In diffusion models, we define the generative process according to the true denoising process. However, for such a denoising process, we do not know the value of $\mathbf{x}$ a priori, so we typically approximate it as $\hat{\mathbf{x}} = \phi(\mathbf{z}_t, t)$ using a neural network ϕ. Doing so then lets us express the generative transition distribution $p(\mathbf{z}_s|\mathbf{z}_t)$ as $q(\mathbf{z}_s|\hat{\mathbf{x}}(\mathbf{z}_t, t), \mathbf{z}_t)$. As a practical alternative to Eq. A12, we can represent this expression using the approximation for $\hat{\mathbf{x}}$:

$$p(\mathbf{z}_s|\mathbf{z}_t) = \mathcal{N}(\mathbf{z}_s|\boldsymbol{\mu}_{t\to s}(\hat{\mathbf{x}}, \mathbf{z}_t), \sigma_{t\to s}^2 \mathbf{I}). \tag{A13}$$

If we choose to define s as $s = t - 1$, then we can derive the variational lower bound on the log-likelihood of $\mathbf{x}$ given the generative model as:

$$\log p(\mathbf{x}) \geq \mathcal{L}_0 + \mathcal{L}_{base} + \sum_{t=1}^{T} \mathcal{L}_t, \tag{A14}$$

where we note that $\mathcal{L}_0 = \log p(\mathbf{x}|\mathbf{z}_0)$ models the likelihood of the data given its noisy representation $\mathbf{z}_0$, $\mathcal{L}_{base} = -\mathrm{KL}(q(\mathbf{z}_T|\mathbf{x})|p(\mathbf{z}_T))$ models the difference between a standard normal distribution and the final latent variable $q(\mathbf{z}_T|\mathbf{x})$, and

$$\mathcal{L}_t = -\mathrm{KL}(q(\mathbf{z}_s|\mathbf{x}, \mathbf{z}_t)|p(\mathbf{z}_s|\mathbf{z}_t)) \quad \text{for } t = 1, 2, ..., T.$$

Note that, in this formation of diffusion models, the neural network ϕ directly predicts $\hat{\mathbf{x}}$. However, Ho et al. [3] and others have found optimization of ϕ to be made much easier when instead predicting the Gaussian noise added to $\mathbf{x}$ to create $\hat{\mathbf{x}}$. An intuition for how this changes the neural network's learning dynamics is that, when predicting back the noise added to the model's input, the network is being trained to more directly differentiate which part of $\mathbf{z}_t$ corresponds to the input's feature signal (i.e., the underlying data point $\mathbf{x}$) and which part corresponds to added feature noise. In doing so, if we let $\mathbf{z}_t = \alpha_t \mathbf{x} + \sigma_t \boldsymbol{\epsilon}$, the neural network can then predict $\hat{\boldsymbol{\epsilon}} = \phi(\mathbf{z}_t, t)$ such that:

$$\hat{\mathbf{x}} = (1/\alpha_t)\ \mathbf{z}_t - (\sigma_t/\alpha_t)\ \hat{\boldsymbol{\epsilon}}. \tag{A15}$$

Kingma et al. [6] and others have since shown that, when parametrizing the denoising neural network in this way, the loss term $\mathcal{L}_t$ reduces to:

$$\mathcal{L}_t = \mathbb{E}_{\boldsymbol{\epsilon} \sim \mathcal{N}(\mathbf{0}, \mathbf{I})} \left[\frac{1}{2} (1 - \mathrm{SNR}(t-1)/\mathrm{SNR}(t)) \| \boldsymbol{\epsilon} - \hat{\boldsymbol{\epsilon}} \|^2 \right] \tag{A16}$$

Note that, in practice, the loss term $\mathcal{L}_{base}$ should be close to zero when using a noising schedule defined such that $\alpha_T \approx 0$. Moreover, if and when $\alpha_0 \approx 1$ and $\mathbf{x}$ is a discrete value, we will find $\mathcal{L}_0$ to be close to zero as well.

A.2.2 Zeroth Likelihood Terms for GCDM Optimization Objective

For the zeroth likelihood terms corresponding to each type of input feature, we directly adopt the respective terms previously derived by Hoogeboom et al. [7]. Doing so enables a negative log-likelihood calculation for GCDM's predictions. In particular, for integer node features, we adopt the zeroth likelihood term:

$$p(\mathbf{h}|\mathbf{z}_0^{(h)}) = \int_{\mathbf{h}-\frac{1}{2}}^{\mathbf{h}+\frac{1}{2}} \mathcal{N}(\mathbf{u}|\mathbf{z}_0^{(h)}, \sigma_0) \mathrm{d}\mathbf{u}, \tag{A17}$$

where we use the CDF of a standard normal distribution, Φ, to compute Eq. A17 as $\Phi((\mathbf{h} + \frac{1}{2} - \mathbf{z}_0^{(h)})/\sigma_0) - \Phi((\mathbf{h} - \frac{1}{2} - \mathbf{z}_0^{(h)})/\sigma_0) \approx 1$ for reasonable noise parameters α_0

and σ_0 [7]. For categorical node features, we instead use the zeroth likelihood term:

$$p(\mathbf{h}|\mathbf{z}_0^{(h)}) = C(\mathbf{h}|\mathbf{p}), \mathbf{p} \propto \int_{1-\frac{1}{2}}^{1+\frac{1}{2}} \mathcal{N}(\mathbf{u}|\mathbf{z}_0^{(h)}, \sigma_0)\mathrm{d}\mathbf{u}, \tag{A18}$$

where we normalize $\mathbf{p}$ to sum to one and where C is a categorical distribution [7]. Lastly, for continuous node positions, we adopt the zeroth likelihood term:

$$p(\mathbf{x}|\mathbf{z}_0^{(x)}) = \mathcal{N}\left(\mathbf{x}|\mathbf{z}_0^{(x)}/\alpha_0 - \sigma_0/\alpha_0\hat{\boldsymbol{\epsilon}}_0, \sigma_0^2/\alpha_0^2\mathbf{I}\right) \tag{A19}$$

which gives rise to the log-likelihood component $\mathcal{L}_0^{(x)}$ as:

$$\mathcal{L}_0^{(x)} = \mathbb{E}_{\boldsymbol{\epsilon}^{(x)} \sim \mathcal{N}_x(\mathbf{0},\mathbf{I})}\left[\log Z^{-1} - \frac{1}{2}\|\boldsymbol{\epsilon}^x - \phi^{(x)}(\mathbf{z}_0, 0)\|^2\right], \tag{A20}$$

where $d = 3$ and the normalization constant $Z = (\sqrt{2\pi} \cdot \sigma_0/\alpha_0)^{(N-1)\cdot d}$ - in particular, its $(N-1) \cdot d$ term - arises from the zero center of gravity trick mentioned in Section 4.4 of the main text [7].

A.2.3 Diffusion Models and Equivariant Distributions

In the context of diffusion generative models of 3D data, one often desires for the marginal distribution $p(\mathbf{x})$ of their denoising neural network to be an invariant distribution. Towards this end, we observe that a conditional distribution $p(y|x)$ is equivariant to the action of 3D rotations by meeting the criterion:

$$p(y|x) = p(\mathbf{R}y|\mathbf{R}x) \quad \text{for all orthogonal } \mathbf{R}. \tag{A21}$$

Moreover, a distribution is invariant to rotation transformations $\mathbf{R}$ when

$$p(y) = p(\mathbf{R}y) \quad \text{for all orthogonal } \mathbf{R}. \tag{A22}$$

As Köhler et al. [8] and Xu et al. [9] have collectively demonstrated, we know that if $p(\mathbf{z}_T)$ is invariant and the neural network we use to parametrize $p(\mathbf{z}_{t-1}|\mathbf{z}_t)$ is equivariant, we have, as desired, that the marginal distribution $p(\mathbf{x})$ of the denoising model is an invariant distribution.

A.2.4 Training and Sampling Procedures for GCDM

Equivariant Dynamics. In this work, we use the previous definition of GCPNet++ in Section A.1.2 to learn an SE(3)-equivariant dynamics function $[\hat{\boldsymbol{\epsilon}}^{(x)}, \hat{\boldsymbol{\epsilon}}^{(h)}] = \phi(\mathbf{z}_t^{(x)}, \mathbf{z}_t^{(h)}, t)$ as:

$$\hat{\boldsymbol{\epsilon}}_t^{(x)}, \hat{\boldsymbol{\epsilon}}_t^{(h)} = \text{GCPNet++}(\mathbf{z}_t^{(x)}, [\mathbf{z}_t^{(h)}, \psi(\mathbf{z}_t^{(x)}), t/T]) - [\mathbf{z}_t^{(x)}, \mathbf{0}], \tag{A23}$$

where we inform the denoising model of the current time step by concatenating t/T as an additional node feature and where we subtract the coordinate representation outputs of GCPNet++ from its coordinate representation inputs after subtracting from the coordinate representation outputs their collective center of gravity. Lastly yet importantly, as a geometric GNN, GCPNet++ can embed geometric vector features in addition to scalar features. Subsequently, from the noisy coordinates representation $\mathbf{z}_t^{(x)}$ we derive noisy sequential (node) orientation unit vectors and pairwise (edge) displacement unit vectors $\psi(\mathbf{z}_t^{(x)})$, respectively, and embed these features using GCPNet++'s vector feature channels for nodes and edges accordingly. With the parametrization in Eq. 5 of the main text, GCDM subsequently achieves rotation equivariance on $\hat{\mathbf{x}}_i$, thereby achieving a 3D translation and rotation-invariant marginal distribution $p(\mathbf{x})$ as described in Appendix A.2.3.

Scaling Node Features. In line with Hoogeboom et al. [7], to improve the log-likelihood of the model's generated samples, we find it useful to train and perform sampling with GCDM using scaled node feature inputs as $[\mathbf{x}, \frac{1}{4}\mathbf{h}^{(categorical)}, \frac{1}{10}\mathbf{h}^{(integer)}]$.

Deriving The Number of Atoms. Finally, to determine the number of atoms with which GCDM will generate a 3D molecule, we first sample $N \sim p(N)$, where $p(N)$ denotes the categorical distribution of molecule sizes over GCDM's training dataset. Then, we conclude by sampling $\mathbf{x}, \mathbf{h} \sim p(\mathbf{x}, \mathbf{h}|N)$.

Appendix B Supplementary Notes

B.1 Broader Impacts

In this work, we investigate the impact of geometric representation learning on generative models for 3D molecules. Such research can contribute to drug discovery efforts by accelerating the development of new medicinal or energy-related molecular compounds, and, as a consequence, can yield positive societal impacts [10]. Nonetheless, in line with Urbina et al. [11], we authors would argue that it will be critical for institutions, governments, and nations to reach a consensus on the strict regulatory practices that should govern the use of such molecule design methodologies in settings in which it is reasonably likely such methodologies could be used for nefarious purposes by scientific "bad actors".

B.2 Training Details

Scalar Message Attention. In our implementation of scalar message attention (SMA) within GCDM, $\mathbf{m}_{ij} = e_{ij}\mathbf{m}_{ij}$, where $\mathbf{m}_{ij}$ represents the scalar messages learned by GCPNet++ during message-passing and e_{ij} represents a 1 if an edge exists between nodes i and j (and a 0 otherwise) via $e_{ij} \approx \phi_{inf}(\mathbf{m}_{ij})$. Here, $\phi_{inf} : \mathbb{R}^e \rightarrow [0,1]^1$ resembles a linear layer followed by a sigmoid function [12].

GCDM Hyperparameters. All GCDM models train on QM9 for approximately 1,000 epochs using 9 **GCPConv** layers; SiLU activations [13]; 256 and 64 scalar node and edge hidden features, respectively; and 32 and 16 vector-valued node and edge features, respectively. All GCDM models are also trained using the AdamW optimizer [14] with a batch size of 64, a learning rate of 10^{-4}, and a weight decay rate of 10^{-12}.

GCDM Runtime. With a maximum batch size of 64, this 9-layer model configuration allows us to train GCDM models for unconditional (conditional) tasks on the QM9 dataset using approximately 10 (15) days of GPU training time with a single 24GB NVIDIA A10 GPU. For unconditional molecule generation on the much larger GEOM-Drugs dataset, a maximum batch size of 64 allows us to train 4-layer GCDM models using approximately 60 days of GPU training time with a single 48GB NVIDIA RTX A6000 GPU. As such, access to several GPUs with larger GPU memory limits (e.g., 80GBs) should allow one to concurrently train GCDM models in a fraction of the time via larger batch sizes or data-parallel training techniques [15].

B.3 Compute Requirements

Training GCDM models for tasks on the QM9 dataset by default requires a GPU with at least 24GB of GPU memory. Inference with such GCDM models for QM9 is much more flexible in terms of GPU memory requirements, as users can directly control how soon a molecule generation batch will complete according to the size of molecules being generated as well as one's selected batch size during sampling. Training GCDM models for unconditional molecule generation on the GEOM-Drugs dataset by default requires a GPU with at least 48GB of GPU memory. Similar to the GCDM models for QM9, inference with GEOM-Drugs models is flexible in terms of GPU memory requirements according to one's choice of sampling hyperparameters. Note that inference for both

QM9 models and GEOM-Drugs models can likely be accelerated using techniques such as DDIM sampling [16]. However, we have not officially validated the quality of generated molecules using such sampling techniques, so we caution users to be aware of this potential risk of degrading molecule sample quality when using such sampling algorithms.

B.4 Reproducibility

On GitHub, we thoroughly provide all source code, data, and instructions required to train new GCDM models or reproduce our results for each of the four protein-independent molecule generation tasks we study in this work. The source code, data, and instructions for our protein-conditional molecule generation experiments are also available on GitHub. Our source code uses PyTorch [17] and PyTorch Lightning [15] to facilitate model training; PyTorch Geometric [18] to support sparse tensor operations on geometric graphs; and Hydra [19] to enable reproducible hyperparameter and experiment management.

Task	$\alpha \downarrow$ / $MS \uparrow$	$\Delta\epsilon \downarrow$ / $MS \uparrow$	$\epsilon_{HOMO} \downarrow$ / $MS \uparrow$	$\epsilon_{LUMO} \downarrow$ / $MS \uparrow$	$\mu \downarrow$ / $MS \uparrow$	$C_v \downarrow$ / $MS \uparrow$
Units	$Bohr^3$ / %	meV / %	meV / %	meV / %	D / %	$\frac{cal}{mol}K$ / %
Initial Samples (Moderately Stable)	4.61 ± 0.2 / 61.7	1.26 ± 0.1 / 61.7	0.53 ± 0.0 / 61.7	1.25 ± 0.0 / 61.7	1.35 ± 0.1 / 61.7	2.93 ± 0.1 / 61.7
EDM-Opt (100 steps on initial samples)	4.45 ± 0.6 / 77.6 ± 2.1	0.98 ± 0.1 / 80.0 ± 2.0	0.45 ± 0.0 / 78.8 ± 1.0	0.91 ± 0.0 / 83.4 ± 4.6	$6e^5 \pm 6e^5$ / 78.3 ± 2.9	2.72 ± 2.6 / 51.0 ± 109.7
EDM-Opt (250 steps on initial samples)	$1e^2 \pm 5e^2$ / 80.1 ± 2.1	$1e^3 \pm 6e^3$ / 83.7 ± 3.8	0.44 ± 0.0 / 82.5 ± 1.3	0.91 ± 0.1 / $\underline{84.7} \pm 1.6$	$2e^5 \pm 8e^5$ / $\underline{81.0} \pm 5.8$	$\underline{2.15} \pm 0.1$ / $\underline{78.5} \pm 3.4$
GCDM-Opt (100 steps on initial samples)	$\underline{3.29} \pm 0.1$ / $\underline{86.2} \pm 1.3$	$\underline{0.93} \pm 0.0$ / $\underline{89.0} \pm 1.9$	$\mathbf{0.43} \pm 0.0$ / $\mathbf{91.6} \pm 3.5$	$\underline{0.86} \pm 0.0$ / $\underline{87.0} \pm 1.7$	$\underline{1.08} \pm 0.1$ / $\mathbf{89.9} \pm 4.2$	$\mathbf{1.81} \pm 0.0$ / $\underline{87.6} \pm 1.1$
GCDM-Opt (250 steps on initial samples)	$\mathbf{3.24} \pm 0.2$ / $\mathbf{86.6} \pm 1.9$	$\mathbf{0.93} \pm 0.0$ / $\mathbf{89.7} \pm 2.2$	$\underline{0.43} \pm 0.0$ / $\underline{90.7} \pm 0.0$	$\mathbf{0.85} \pm 0.0$ / $\mathbf{88.6} \pm 3.8$	$\mathbf{1.04} \pm 0.0$ / $\underline{89.5} \pm 2.6$	$\underline{1.82} \pm 0.1$ / $\mathbf{87.6} \pm 2.3$

Table C1: **Comparison of GCDM with baseline methods for property-guided 3D molecule optimization.** The results are reported in terms of molecular stability (MS) and the MAE for molecular property prediction by an ensemble of three EGNN classifiers ϕ_c (each trained on the same QM9 subset using a distinct random seed) yielding corresponding Student's t-distribution 95% confidence intervals, with results listed for EDM and GCDM-optimized samples as well as the molecule generation baseline ("Initial Samples"). Note that certain experiments with an EDM optimizer yielded unsuccessful property optimization, where we denote such results as outlier property MAE values greater than 50. The top-1 (best) results for this task are in **bold**, and the second-best results are underlined.

Appendix C Supplementary Results

C.1 Property-Guided 3D Molecule Optimization - QM9

In Table C1, for completeness, we list the numeric molecule optimization results comprising Figure 6 of the main text.

References

[1] Du, W., Zhang, H., Du, Y., Meng, Q., Chen, W., Zheng, N., Shao, B., Liu, T.-Y.: SE(3) equivariant graph neural networks with complete local frames. In: Chaudhuri, K., Jegelka, S., Song, L., Szepesvari, C., Niu, G., Sabato, S. (eds.) Proceedings of the 39th International Conference on Machine Learning. Proceedings of Machine Learning Research, vol. 162, pp. 5583–5608 (2022)

[2] Morehead, A., Cheng, J.: Geometry-complete perceptron networks for 3d molecular graphs. Bioinformatics (2024)

[3] Ho, J., Jain, A., Abbeel, P.: Denoising diffusion probabilistic models. Advances in Neural Information Processing Systems **33**, 6840–6851 (2020)

[4] Joshi, C.K., Bodnar, C., Mathis, S.V., Cohen, T., Liò, P.: On the expressive power of geometric graph neural networks. arXiv preprint arXiv:2301.09308 (2023)

[5] Sohl-Dickstein, J., Weiss, E., Maheswaranathan, N., Ganguli, S.: Deep unsupervised learning using nonequilibrium thermodynamics. In: International Conference on Machine Learning, pp. 2256–2265 (2015). PMLR

[6] Kingma, D., Salimans, T., Poole, B., Ho, J.: Variational diffusion models. Advances in neural information processing systems **34**, 21696–21707 (2021)

[7] Hoogeboom, E., Satorras, V.G., Vignac, C., Welling, M.: Equivariant diffusion for molecule generation in 3d. In: International Conference on Machine Learning, pp. 8867–8887 (2022). PMLR

[8] Köhler, J., Klein, L., Noé, F.: Equivariant flows: exact likelihood generative learning for symmetric densities. In: International Conference on Machine Learning, pp. 5361–5370 (2020). PMLR

[9] Xu, M., Yu, L., Song, Y., Shi, C., Ermon, S., Tang, J.: Geodiff: A geometric diffusion model for molecular conformation generation. arXiv preprint arXiv:2203.02923 (2022)

[10] Walters, W.P., Murcko, M.: Assessing the impact of generative ai on medicinal chemistry. Nature biotechnology **38**(2), 143–145 (2020)

[11] Urbina, F., Lentzos, F., Invernizzi, C., Ekins, S.: Dual use of artificial-intelligence-powered drug discovery. Nature Machine Intelligence **4**(3), 189–191 (2022)

[12] Satorras, V.G., Hoogeboom, E., Welling, M.: E (n) equivariant graph neural networks. In: International Conference on Machine Learning, pp. 9323–9332 (2021). PMLR

[13] Elfwing, S., Uchibe, E., Doya, K.: Sigmoid-weighted linear units for neural network function approximation in reinforcement learning. Neural Networks **107**,

3–11 (2018)

[14] Loshchilov, I., Hutter, F.: Decoupled weight decay regularization. arXiv preprint arXiv:1711.05101 (2017)

[15] Falcon, W.A.: Pytorch lightning. GitHub **3** (2019)

[16] Song, J., Meng, C., Ermon, S.: Denoising diffusion implicit models. arXiv preprint arXiv:2010.02502 (2020)

[17] Paszke, A., Gross, S., Massa, F., Lerer, A., Bradbury, J., Chanan, G., Killeen, T., Lin, Z., Gimelshein, N., Antiga, L., et al.: Pytorch: An imperative style, high-performance deep learning library. Advances in neural information processing systems **32** (2019)

[18] Fey, M., Lenssen, J.E.: Fast graph representation learning with pytorch geometric. arXiv preprint arXiv:1903.02428 (2019)

[19] Yadan, O.: Hydra - A framework for elegantly configuring complex applications. Github (2019). https://github.com/facebookresearch/hydra
